# Etiologies of chronic cough in children: a two-year experience from a tertiary pediatric pulmonology center

**DOI:** 10.55730/1300-0144.6151

**Published:** 2025-12-20

**Authors:** Yasin Maruf ERGEN, Nagehan EMİRALİOĞLU, Ebru YALÇIN, Deniz DOĞRU, Uğur ÖZÇELİK, Nural KİPER

**Affiliations:** 1 Department of Pediatrics, Faculty of Medicine, Hacettepe University, Ankara, Turkiye; 2 Department of Pediatric Pulmonology, Faculty of Medicine, Hacettepe University, Ankara, Turkiye

**Keywords:** Children, chronic cough, wet cough, asthma, primary ciliary dyskinesia

## Abstract

**Background/aim:**

Chronic cough is a common yet diagnostically challenging symptom in pediatric pulmonology. This study aimed to evaluate the etiologies of chronic cough in children referred to a tertiary center and to analyze the relationship between specific cough characteristics and final diagnoses.

**Materials and methods:**

This retrospective study evaluated patients presenting with chronic cough (duration >4 weeks) at a tertiary pediatric pulmonology center. Demographic data, cough characteristics (wet vs. dry), and diagnostic findings were analyzed.

**Results:**

A total of 62 patients were included. A specific etiology was identified in 95.1% of the patients. Asthma and reactive airway disease were the most common diagnoses (45.2%), predominantly associated with dry cough. Notably, we observed an unexpectedly high prevalence of primary ciliary dyskinesia (PCD) (19.4%) and cystic fibrosis (CF) (6.5%), particularly in the wet cough group. This distribution differs significantly from primary care studies, reflecting the selected, refractory nature of patients referred to our tertiary center. Protracted bacterial bronchitis (PBB) was identified in only 3.2% of cases.

**Conclusion:**

While asthma remains the leading cause of dry cough, structural lung diseases such as PCD and CF are major etiologies in children presenting with chronic wet cough in tertiary settings. The high rate of these serious conditions underscores the need for early and detailed investigation in patients unresponsive to standard therapies, rather than repeated empirical treatments for presumed PBB.

## 1. Introduction

A cough is a crucial reflex that protects the lungs from foreign substances, particles, secretions, and mucus that may be inhaled. The absence of this mechanism can be fatal for human survival [1]. It is a common reason for hospital visits and a major source of distress for both children and parents [2]. Repeated hospital visits may result in unnecessary tests and excessive medication use. A cohort study in the United Kingdom found that an average of £3663 was spent per patient with chronic cough per year [3,4].

Defining chronic cough in children remains a subject of debate due to varying guideline recommendations. The American College of Chest Physicians utilizes a cutoff of 4 weeks [5]. In contrast, the European Respiratory Society defines chronic cough as lasting longer than 8 weeks to exclude postviral etiologies [6]. From a clinical perspective, the 4-week definition adopted in this study enables earlier identification of underlying pathologies, such as protracted bacterial bronchitis (PBB), thereby preventing the potential progression of suppurative lung disease. This distinction is crucial, as healthy children may cough for up to three weeks following a viral infection [7]. However, persistence beyond 4 weeks warrants investigation, as a multicenter study reported that 17.6% of such children had serious lung diseases, including bronchiectasis, aspiration, and cystic fibrosis [8]. The most common causes of chronic cough in children are asthma, PBB, and upper airway cough syndrome [9].

We hypothesized that the etiological profile of chronic cough in children referred to a tertiary care center differs significantly from primary care cohorts, characterized by a higher prevalence of structural lung diseases rather than simple infections. Therefore, this study aimed to evaluate the specific etiologies of chronic cough in a tertiary pediatric pulmonology setting and to determine how cough character (wet vs. dry) guides the differential diagnosis toward specific conditions, such as primary ciliary dyskinesia (PCD) and cystic fibrosis (CF).

## 2. Materials and methods

### 2.1. Patients

**Figure 1:**
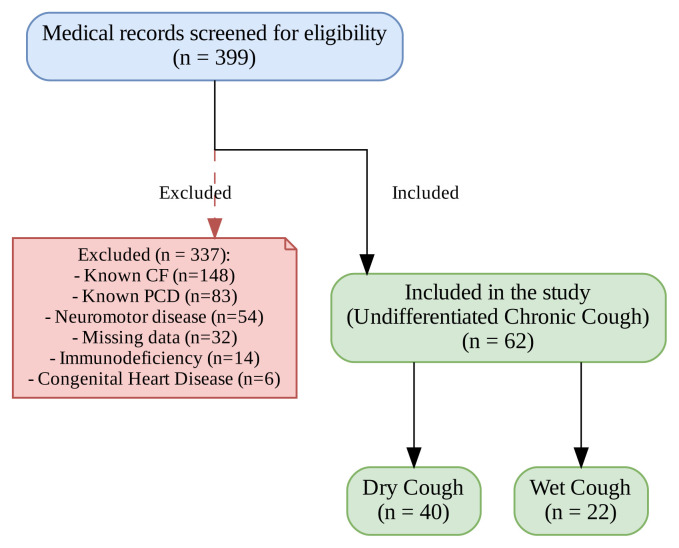
Study flow diagram illustrating the screening process, exclusion criteria, and the final study population.

All consecutive children aged 1 month to 18 years who presented with chronic cough to the Pediatric Pulmonology Department at Hacettepe University Faculty of Medicine, a tertiary care center, between July 2015 and July 2017, were retrospectively evaluated. A cough lasting longer than 4 weeks was defined as chronic [10]. To focus on the etiological evaluation of undifferentiated chronic cough, patients with a previously established diagnosis of chronic respiratory or systemic disorders prior to referral-including cardiac disease, neuromotor developmental delay, swallowing dysfunction, immunodeficiency, or chronic lung diseases such as CF, PCD, and bronchiectasis-were excluded from the analysis. The detailed patient selection algorithm and exclusion process are illustrated in Figure 1.

### 2.2. Study design

Demographic data (age, sex), cough characteristics, associated symptoms, medication use, family history, cigarette smoke exposure, and history of tuberculosis contact were retrospectively extracted from the patients’ medical records. Clinical management and diagnostic evaluations had been carried out during routine care in accordance with the recommendations of the American College of Chest Physicians [10]. Specific cough markers documented by clinicians in accordance with guideline definitions included wet cough, digital clubbing, failure to thrive, chest wall deformity, and recurrent respiratory infections.

Asthma diagnosis was based on the Global Initiative for Asthma (GINA) guidelines. For children over 6 years, diagnosis was confirmed by reviewing spirometry records for significant reversibility (>12% increase in FEV1) [11]. In children <6 years, the diagnosis was defined by documented recurrent wheezing, a positive Modified Asthma Predictive Index (mAPI), and clinical improvement following a therapeutic trial of inhaled corticosteroids [12].

Data regarding diagnostic procedures were extracted from patient medical records. All diagnostic tests were performed as part of standard routine clinical care in accordance with international guidelines. Pediatric pulmonologists evaluated chest radiographs and pulmonary function tests (PFTs) during the clinic visits. Flexible bronchoscopy and high-speed video microscopy (HSVM) were performed and assessed by pediatric pulmonologists for microbiological sampling, anatomical evaluation, or ciliary function analysis, as indicated. Pediatric radiologists interpreted high-resolution computed tomography (HRCT) scans as part of the routine diagnostic workflow, and these official radiology reports were utilized for the study analysis.

All patients had undergone chest radiography, and PFT by spirometry was performed when age-appropriate. In patients recorded to have specific cough markers, available results of hemogram, liver and kidney function tests, quantitative immunoglobulin levels (IgG, IgA, IgM, IgE), Mycoplasma pneumoniae antibody test, tuberculin skin test [purified protein derivative (PPD)], sweat chloride test, and sputum culture were retrieved from the hospital database.

Advanced diagnostic procedures had been performed based on guideline-supported clinical indications documented in the patient files: HSVM was performed in patients with persistent wet cough or neonatal respiratory distress suggestive of primary ciliary dyskinesia; thoracic HRCT was obtained in patients with persistent radiological abnormalities or recurrent lower respiratory tract infections; and flexible bronchoscopy was performed in patients with suspected foreign body aspiration or nonresponse to therapy [10,13].

Referral records to pediatric allergy, gastroenterology, and otorhinolaryngology specialists were also reviewed when reactive airway symptoms, gastroesophageal reflux, or upper airway cough syndrome were suspected. Examination processes, final diagnoses, and follow-up data for at least 6 months were collected retrospectively from electronic medical records.

### 2.3. Statistical analysis

Statistical analysis was performed using SPSS version 22.0. Categorical variables were presented as frequencies and percentages. Comparisons between groups were performed using the chi-square test or Fisher’s exact test. Univariate logistic regression was used to estimate odds ratios (ORs) with 95% confidence intervals (CIs) to assess the strength of associations between clinical features (e.g., wet cough, digital clubbing) and final diagnoses. A p-value of <0.05 was considered statistically significant.

### 2.4. Ethical approval

This study was approved by the Ethics Committee of Hacettepe University (Approval number: GO 17/721-27; September 2017).

## 3. Results

### 3.1. Demographics

A total of 399 medical records were screened. Following the exclusion of 337 patients due to previously established diagnoses or incomplete data. The study flow diagram is presented in Figure 1.

A total of 62 patients (34 males, 54.9%; 28 females, 45.1%) were included in the study. The mean age of the patients was 6.30 ± 4.30 years (range: 3 months–16.1 years). Parental consanguinity was observed in 14 (22.5%) of the families. A family history of PCD was noted in three patients, and CF in one patient. The frequency of admissions showed seasonal variation, peaking in December (n=13, 21%) and reaching its lowest point during the summer months (July and August).

Smoke exposure was present in 13 (20.9%) patients. No statistically significant association was observed between tobacco exposure and the diagnosis of any chronic disease (p > 0.05).

A history of contact with tuberculosis was positive in only two patients; one was diagnosed with active tuberculosis, confirming the importance of contact history, while the other was diagnosed with PBB.

#### 3.1.1. Cough characteristics

Cough persisted for more than 3 months in 75.8% of patients. The most common associated symptom was wheezing (67.7%). Regarding cough character, 40 patients (64.5%) presented with dry cough, while 22 patients (35.5%) had wet cough.

Specific clinical pointers were noted in several patients; notably, digital clubbing was present in five patients, and recurrent lower respiratory tract infections were noted in 15 patients (24.1%). Two patients specifically reported no coughing during sleep, suggestive of psychogenic cough.

The presence of specific cough pointers significantly increased the diagnostic yield for structural lung diseases. Notably, digital clubbing was found to be a highly specific marker; 100% of patients presenting with digital clubbing were diagnosed with either CF or PCD. Conversely, all patients (n=3) in whom a specific etiology could not be identified (unknown diagnosis) presented with isolated dry cough without any specific pointers.

#### 3.1.2. Diagnostic evaluation

Routine laboratory investigations, including hemogram and liver/kidney function tests, were normal in all patients. Hypogammaglobulinemia was detected in four patients. Although multidisciplinary consultations were performed (otorhinolaryngology, n = 21; pediatric gastroenterology, n = 9), no primary otorhinolaryngologic or gastrointestinal pathologies were identified.

Chest radiography was normal in 30 (53.2%) patients, with atelectasis being the most common abnormality (24.2%). Thoracic CT was performed in 21 patients; predominant findings included atelectasis (n = 12) and bronchiectasis (n = 7). Structural anomalies, including a left subclavian artery anomaly, dextrocardia, and bronchiolitis obliterans, were observed in single cases.

Specific diagnostic testing revealed positive sweat chloride levels (>60 mmol/L) in three patients and a positive tuberculin skin test (20 mm) in one patient. Ciliary analysis via high-speed video microscopy was abnormal in 12 (19.4%) patients.

Flexible bronchoscopy was performed on 18 patients. Diffuse purulent secretion was the most common macroscopic finding (n = 7). Other structural findings included tracheomalacia (n = 2), foreign body aspiration (n = 1), hydatid cyst (n = 1), and external tracheal compression (n = 1). Analysis of bronchoalveolar lavage (BAL) fluid revealed lipid-laden macrophages in three patients. BAL cultures yielded *Streptococcus pneumoniae* (n = 3), *Haemophilus influenzae* (n = 2), and *Pseudomonas aeruginosa* (n = 1).

### 3.2. Final diagnoses

**Figure 2:**
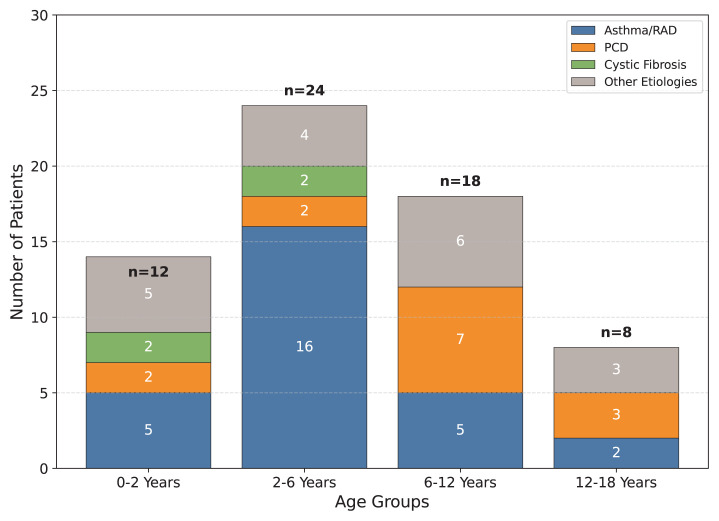
Distribution of final diagnoses stratified by developmental age groups. Asthma/reactive airway disease was the predominant etiology in the preschool age group (2–6 years), whereas the frequency of PCD increased in older children.

Of the 62 patients admitted with chronic cough, an etiology was identified in 59 patients (95.1%). The distribution of final diagnoses across developmental age groups is shown in Figure 2. Asthma and reactive airway disease were the most frequent diagnoses, identified in 28 patients with a mean age of 4.94 ± 3.44 years. Transient hypogammaglobulinemia was incidentally detected in 3 patients within this group, which resolved during follow-up. Regarding treatment response, cough severity and frequency decreased significantly in all patients following the initiation of inhaler treatment. A critical finding in this cohort was the high rate of prior misdiagnosis; 18 out of 34 patients (52.9%) who were ultimately diagnosed with nonasthmatic conditions had previously received salbutamol due to a suspicion of asthma prior to referral.

PCD was diagnosed in 12 patients (mean age: 9.21 ± 4.07 years). Clinically, this group was distinguished by high rates of recurrent pneumonia (83.3%) and a history of neonatal intensive care admission (41.6%). The diagnosis was definitively confirmed via genetic analysis and high-speed video microscopy in all patients. Regarding supportive testing, nasal nitric oxide (nNO) levels were low (<77 nL/min) in all cooperative patients, and thoracic CT imaging revealed significant structural changes, including middle lobe atelectasis (75%) and extensive bronchiectasis (50%).

The CF cohort (n = 4) represented the youngest diagnostic group (mean age: 2.85 ± 2.77 years), with all patients presenting with cough since infancy. Diagnosis was confirmed genetically in all cases. Notably, one patient had normal sweat chloride levels but carried a CF-causing mutation. Two patients were diagnosed late due to the absence of a national newborn screening program at the time of their birth.

The diagnoses categorized as “Other Etiologies” in Figure 2 included postinfectious cough (n = 3), PBB (n = 2), and psychogenic cough (n = 2). Notably, both patients with PBB achieved a complete cough-free status following a 2-week course of amoxicillin-clavulanate. Psychogenic cough, in contrast, was diagnosed based on the disappearance of symptoms during sleep and a normal diagnostic workup; these cases resolved spontaneously without pharmacotherapy. Rare etiologies identified as single cases (n = 1 each) included vascular ring, lymphoid interstitial pneumonia, foreign body aspiration, Bruton’s disease, tracheomalacia, hydatid cyst, tuberculosis, and bronchiolitis obliterans. In three patients, the etiology remained unknown despite detailed evaluation.

#### 3.2.1. Etiological distribution by cough character (wet vs. dry)

**Figure 3:**
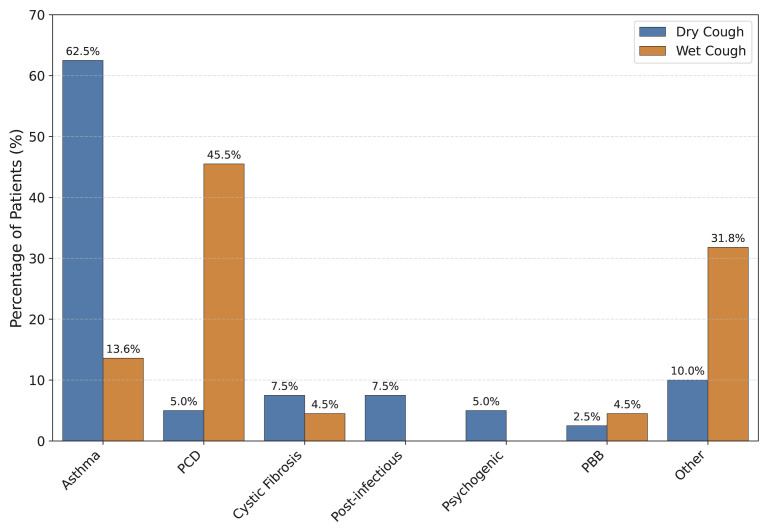
Comparative distribution of etiologies according to cough character (wet vs. dry). Asthma was significantly more prevalent in the dry cough group (62.5%), while PCD was strongly associated with wet cough (45.5%). PCD: primary ciliary dyskinesia, PBB: protracted bacterial bronchitis.

The study population was divided into two groups based on cough character, and the distribution of diagnoses is presented in Figure 3.

Among the 40 patients with dry cough, asthma, and reactive airway disease were the predominant etiologies. In patients old enough to perform spirometry, reversible obstruction was observed in pulmonary function tests.

In contrast, among the 22 patients with wet cough, PCD was the most common etiology. Statistical analysis revealed that a wet cough was a strong predictor of PCD; children with a wet cough had a 15.8-fold higher risk of PCD than those with a dry cough (OR: 15.8, 95% CI: 3.1–80.2, p < 0.001).

## 4. Discussion

In this study, consistent with other studies from Türkiye [15,16,17], asthma and reactive airway disease were the most common diagnoses. However, our findings diverged from international reports regarding other etiologies. For instance, PBB is often reported as the leading cause in Australian studies [8,18]. In contrast, infections or upper airway cough syndrome predominate in reports from the USA and China [14,19]. We attribute these discrepancies primarily to referral bias inherent to the tertiary nature of our center. Unlike primary care settings, where PBB and upper airway conditions are frequently managed, our clinic receives a selected population enriched for complex pathologies. Consequently, the prevalence of PCD (19.4%) and CF (6.5%) in our cohort was notably high. This elevation is further supported by our access to advanced diagnostic capabilities (e.g., nNO measurement, genetic sequencing), which have minimized missed diagnoses and are epidemiologically consistent with the high rate of parental consanguinity (22.5%) observed in our population. When analyzed by cough character, asthma was the predominant cause in the dry cough group (62.5%). Most guidelines recommend spirometry and bronchodilator reversibility testing to confirm the diagnosis [20]. However, the inability to perform spirometry in children younger than 6 years further complicates this process, often leading to empirical treatment. In our study, we observed that a significant proportion of patients eventually diagnosed with other etiologies (e.g., PCD) had previously received asthma-specific treatment without clinical improvement. This aligns with literature suggesting that asthma is often overdiagnosed in children with nonspecific respiratory symptoms due to diagnostic challenges [21,22]. Therefore, while asthma is the primary consideration for dry cough, the lack of response to inhaled corticosteroids should prompt an immediate investigation for alternative diagnoses, particularly in patients with wet cough.

In the wet cough group, PCD was the leading etiology (45.5%) in our series, ranking second overall after asthma. We acknowledge that the prevalence of PCD in our cohort is notably higher than reported in many other chronic cough studies. This likely reflects the tertiary nature of our clinic, which serves as a reference center for patients with persistent wet cough who are unresponsive to empiric treatments for common etiologies such as PBB. Given the significant symptom overlap with other conditions, PCD can be challenging to identify without specific testing. The mean age of diagnosis in our PCD patients was 9.2 years, which is significantly delayed compared with international data (3.5–8.1 years) [23,24]. Therefore, clinicians must maintain a high index of suspicion for PCD in children with chronic wet cough, neonatal respiratory distress, or bronchiectasis, as early diagnosis is crucial to prevent irreversible lung damage.

CF was the third most common cause overall. The diagnostic gap between screened (5 months) and unscreened (63 months) patients in our study underscores the critical role of newborn screening. Previous studies demonstrate that significant airway inflammation and infection are often present in infants with CF even before the onset of overt symptoms [25]. Consequently, although early diagnosis through screening is ideal to prevent irreversible damage, our findings confirm that CF remains a vital differential diagnosis for older children presenting with chronic wet cough in unscreened populations.

Surprisingly, PBB was identified less frequently (3.2%) in our cohort than reported in the literature [8,18]. We attribute this to the fact that uncomplicated PBB is typically managed successfully in primary care settings. Consequently, patients referred to our tertiary center represent a refractory group where underlying pathologies are more likely than simple PBB. Therefore, to support a crucial practice change, we strongly emphasize that a wet cough unresponsive to a standard 2–4 week antibiotic course must trigger an early and detailed evaluation for structural and motility disorders (PCD, CF) rather than repeated empiric treatment. Similarly, gastroesophageal reflux and upper airway cough syndrome were not identified as primary etiologies, likely because other specialists effectively manage these common conditions before referral to our department.

The presence of specific cough pointers (e.g., wet cough, clubbing) guided us to a diagnosis in 95% of cases, supporting the algorithmic approach recommended by guidelines [5,10]. Conversely, for the minority with isolated dry cough and normal examination, a ‘wait-and-see’ approach prevented unnecessary testing, consistent with reports of spontaneous resolution in such cases [7,13].

Regarding the definition of chronic cough, we advocate for a >4-week cutoff rather than >8 weeks [5,6]. In our cohort, 19.3% (n = 12) of patients presented with a duration of 4–8 weeks. Crucially, applying an 8-week threshold would have delayed the diagnosis of treatable conditions in this group, including PBB and foreign body aspiration. Therefore, we believe a 4-week limit is safer for early detection.

This study has several limitations inherent to its design. First, the retrospective, single-center design and relatively small sample size limit the generalizability of our findings. Second, due to the retrospective design, diagnostic evaluations were not fully standardized for every patient; invasive procedures such as bronchoscopy or high-speed video microscopy were performed only when clinically indicated, rather than as part of a uniform protocol. Finally, the study was conducted in a tertiary reference center, which introduces a significant referral bias, as our cohort consisted largely of complex cases refractory to primary care management.

However, these factors also represent the study’s primary strengths. Unlike primary care studies, our cohort reflects the challenging patient profile encountered by pediatric pulmonologists in specialized centers. Furthermore, despite the lack of a prospective protocol, our comprehensive diagnostic approach-including genetic analysis and advanced ciliary imaging-allowed us to identify a specific etiology in 95.1% of cases. This high diagnostic yield suggests that incorporating an ‘early-referral step’ into clinical algorithms for treatment-refractory wet cough is highly effective in identifying specific etiologies in complex tertiary care patients.

**Figure 4:**
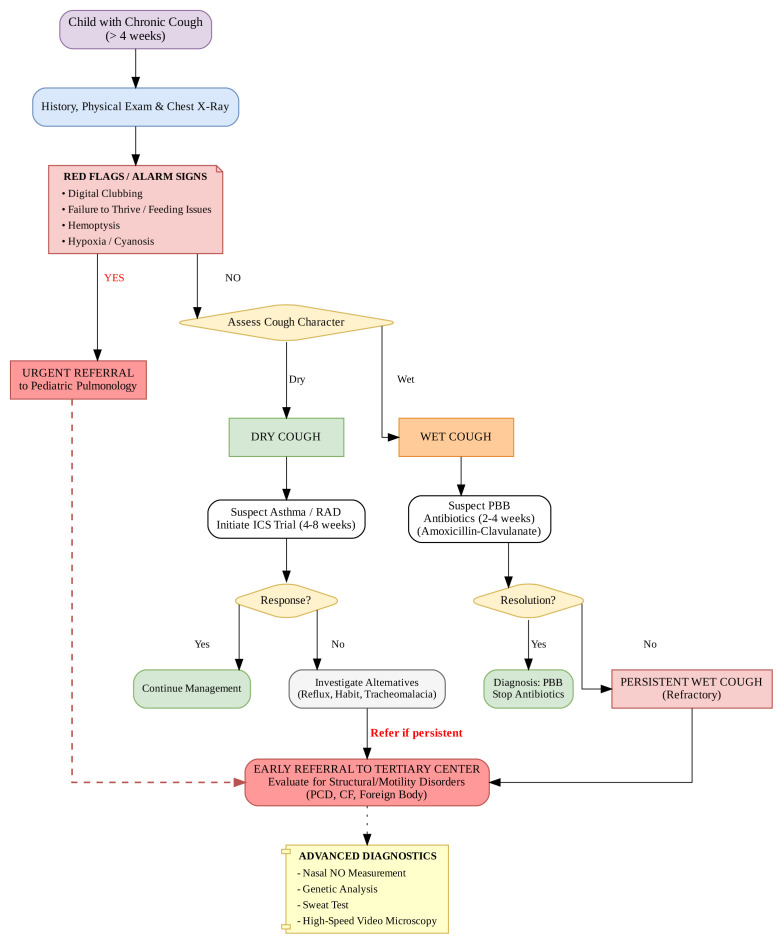
Proposed clinical management algorithm for chronic cough in children. The algorithm integrates specific “Red Flags” (e.g., clubbing) for immediate referral and emphasizes that a wet cough refractory to standard antibiotic therapy (PBB treatment) necessitates early referral to a tertiary center for advanced evaluation (PCD, CF), rather than repeated empiric cycles. PBB: Protracted bacterial bronchitis, RAD: Reactive airway disease, ICS: Inhaled corticosteroids.

In conclusion, our study demonstrates that while asthma is the most frequent cause of chronic cough, tertiary referral centers manage a significantly high burden of complex structural disorders like PCD and CF. Therefore, differentiating between wet and dry cough is the most critical step in the initial evaluation. Crucially, a chronic wet cough that is unresponsive to a standard 2–4 week course of antibiotics (PBB protocol) should be considered a “red flag” for structural airway diseases. To address this, we propose a clinical management algorithm, detailed in Figure 4, that mandates early referral to a tertiary center for such refractory cases, thereby minimizing diagnostic delays by avoiding repeated empiric treatments. Furthermore, in settings with high parental consanguinity, access to advanced diagnostic tools, such as nNO and genetic sequencing, is essential to definitively identify autosomal recessive conditions, such as PCD and CF.
